# Synchronized oscillations of carbon nanotubes dispersed in solution

**DOI:** 10.1038/s41598-023-31813-3

**Published:** 2023-03-20

**Authors:** Makoto Fukumoto, Ryunosuke Akai, Yume Yoshida, Shin-nosuke Sakuma, Hayato Ono, Rintaro Mori, Masahito Sano

**Affiliations:** Department of Organic Materials Science, Yamagata University, 4-3-16 Jyonan, Yonezawa, Yamagata 992-8510 Japan

**Keywords:** Carbon nanotubes and fullerenes, Nonlinear phenomena

## Abstract

Although synchronized oscillations are found in a variety of systems and living organisms in nature, there has been no report on technologically important materials. We have observed by a fluorescence microscope that a large number of carbon nanotubes (CNTs) dispersed in an aqueous mixture of the surfactant and dye execute synchronized oscillations spontaneously. The movement was quantified to give a power spectrum, revealing a single, sharp synchronization peak at 20 Hz. It was found not to be affected nor created by external vibrations. The surfactant concentration dependence demonstrates that the Kuramoto model is applicable to describe the CNT synchronization. It is always associated with the power-law noise, indicating the presence of complex heterogeneous networks. These results suggest a highly cooperative form of the sparse CNT network connected with variable linkages.

## Introduction

Carbon nanotubes (CNTs) have been the subject of intense studies for over 20 years to understand and apply their excellent mechanical, thermal, and electric properties^1,2^. For practical applications, a single CNT is rarely used, and their collective behavior becomes important. Percolation which focuses on the number of connected paths to form an infinite cluster is the most widely accepted model of CNT networks to explain the transport properties of CNT ensemble^3,4^. Also, CNTs were viewed as entangled stiff polymers to analyze some rheological properties^5^. Here, we report that a huge number of CNTs dispersed in a solution spontaneously execute synchronized oscillations (SOs), which implies the existence of a highly cooperative form of the CNT network that has never been recognized. The present finding offers a new concept of self-organized outputs from the CNT network made of finite clusters, resembling neural networks in brains.

SO is a self-organized dynamical process in which a large number of units oscillate with a common frequency. It may occur spontaneously as a result of mutual interactions or may be forced by external stimuli. Some recent examples of qualitatively analyzed synchronization are clocks^6^, frog choruses^7^, and firefly flashes^8^. Synchronization may also arise in chaotic systems^9^. Because various kinds of non-linear interactions, different categories of oscillating units, and complex networks are involved, real systems are mathematically abstracted to tackle theoretical analyses. Graph theory on a topologically abstracted network is an example^10^. In this study, we consider a CNT network cluster as a limit cycle oscillator and compare the experimental results with the results from phase reduction analysis^11^.

**Figure 1:**
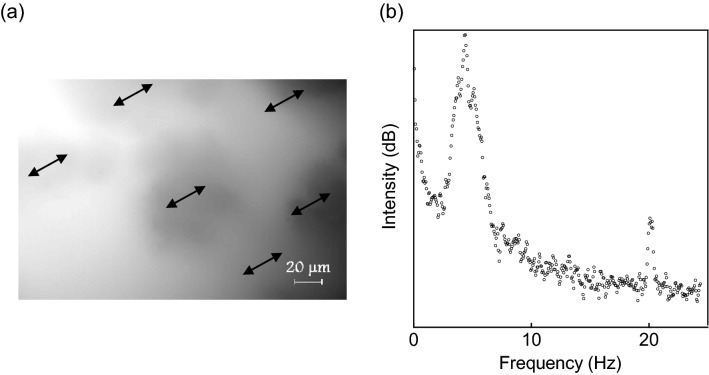
Synchronized oscillation and its power spectrum. (**a**) Fluorescence microscopic image of a CNT dispersion executing SO. The arrows indicate the instantaneous movement. (**b**) Power spectrum of the optical center-of-mass of the shaking image.

We have developed a novel fluorescence microscope that is capable of imaging individual single-layer graphene flakes floating in a solution^12,13^. It uses fluorescence light from the dye dissolved in the dispersing solution as an illumination for the microscope. When it was applied to a CNT dispersion, although individual CNT (diameter 15 nm, length 5–20 μm) was not resolved, slightly dense regions appeared like dark clouds in a brighter background of the less dense region (CNTs were present everywhere in the solution). These clouds float slowly across the viewing window at a good dispersing condition. When the dispersing condition is moderately poor, all clouds oscillate synchronously so that the whole viewing window appears to be shaking (Fig. 1a, see also the Video in Supplementary information). The experiments started with the quantification of the shaking image. The surfactant concentration dependence was investigated since it represented the interaction strength used in the theory. Then, the effect of external vibration and the noise characteristics were studied to elucidate the CNT network that makes SO possible.

## Results

### Power spectrum

Because the entire image shakes, no feature can be used as a reference point to follow the movement. To quantify the oscillation, the real image was formed directly on a 2D position-sensitive detector, which returned the (x, y) coordinate of the optical center-of-mass (CM weighted by the local light intensity *I(x,y;t)*) at time* t*.

1 $${\overrightarrow{r}}_{OCM}\left(t\right)= \frac{\sum_{x,y}I\left(x,y;t\right)\overrightarrow{r}\left(x,y\right)}{\sum_{x,y}I\left(x,y;t\right)}$$

This analog signal was FFT in real time to yield the power spectrum (Fig. 1b). The large peak around 4 Hz is due to the microscope body and is always present regardless of the sample. SO appears as a single sharp peak at approximately 20 Hz. There were no other peaks over the frequency range up to 3 kHz, the frequency bandwidth of the detector.

### Kuramoto model

**Figure 2:**
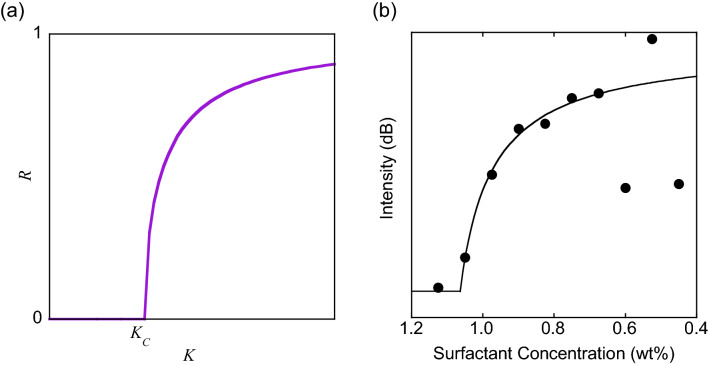
Kuramoto model and surfactant concentration dependence. (**a**) The Kuramoto model predicts the existence of a threshold strength and the square root dependence. (**b**) The SO peak intensity is plotted against the surfactant concentration in descending order. The intensity level at 1.15 wt% is the baseline. The curve is the best fit to the Kuramoto model.

Synchronization is treated theoretically in a framework of limit-cycle oscillations^11^. In particular, the Kuramoto model has been studied intensively since it can be solved analytically^14,15^. The interaction governing the time development of the j-th oscillator phase ${\phi }_{j}$ is given by the sine of the phase differences with others.

2 $$\frac{\mathrm{d}{\phi }_{j}}{\mathrm{dt}}= {\omega }_{j}+ \frac{K}{N}\sum_{k=1}^{N}sin\left({\phi }_{k}-{\phi }_{j}\right)$$

where ${\omega }_{j}$ is the natural frequency, $K$ is the mean-field interaction strength, and $N$ is the number of interacting units. By introducing an order parameter $R$, expressing the phase coherence, by

3 $$R{e}^{i\Theta }= \frac{1}{N}\sum_{j=1}^{N}{e}^{i{\phi }_{j}}$$

and initial phase distribution, it can be shown (for $N\to \infty$) that a threshold strength ${K}_{c}$ exists to synchronize, and that $R$ increases as

4 $$R= \sqrt{\frac{K-{K}_{c}}{K}}$$

as shown in Fig. 2a.

### Surfactant concentration dependence

Because van der Waals (VDW) attractions always act on every CNT^16^, CNTs aggregate rapidly in the solution. To reduce this attraction, a non-ionic surfactant is added. The surfactant molecules are adsorbed spontaneously on the CNT surface. When the surfactant concentration exceeds a critical surface aggregation concentration, the CNT surface is sufficiently covered by the adsorbed molecules so that neighboring CNTs cannot come close to each other. This steric effect keeps the VDW attraction small, and the dispersion becomes stable. In other words, at a concentration below the critical concentration, some parts of the CNT surface are free of the surfactant layer. At the exposed area, neighboring CNTs can approach close enough that the VDW attraction becomes large. Also, it is known that the dye (rhodamine 6G) molecules are adsorbed on the free CNT surfaces^17,18^. Moreover, they tend to self-aggregate in the solution at high concentrations. Thus, neighboring CNTs can be bridged by the adsorbed dye molecules at the exposed area. In this study, the dye concentration is high (250 μmole/L), so the dye-bridging effect may be significant. Both the VDW interaction and the dye-bridging effect take place in the surfactant-free area. Therefore, lowering the surfactant concentration effectively increases the attractive interaction between CNTs and the number of interacting CNTs.

Figure 2b shows the surfactant concentration dependence of SO. The synchronized frequency remains the same over this range. At high concentrations, the VDW attraction is negligible, and the dispersion is stable. No SO is detected. As the concentration decreases, SO suddenly appears at around 1.0 wt% and grows stronger. The dispersion becomes unstable around 0.5–0.6 wt% and forms large aggregates below 0.4 wt%.

The existence of a threshold concentration agrees with the Kuramoto model. Because the phase coherence *R* and the interaction strength *K* are the parameters in the purely mathematical model, their exact relationships to the peak intensity and the surfactant concentration are not known. We think that the agreement between the experimental data and Eq. (4) in Fig. 2b is better-than-expected. Nevertheless, all repeated experiments have yielded concave down, increasing curves, indicating that the Kuramoto model can be applicable to the CNT SO by making a suitable transformation of *K*. It also demonstrates that the peak intensity can be used as a measure of synchronization.

### External vibrations

**Figure 3:**
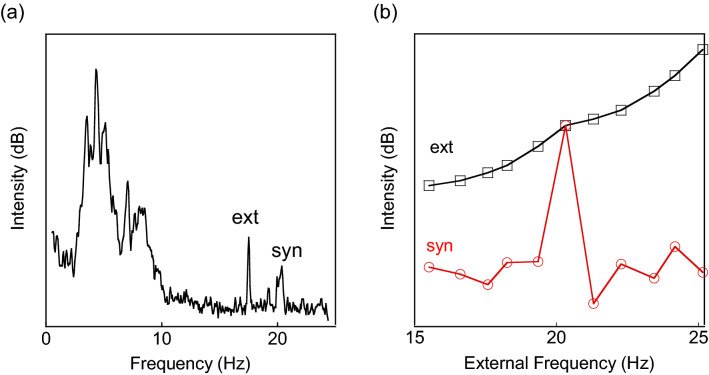
External vibrations. (**a**) A power spectrum when an external vibration is applied at “ext” frequency to a dispersion executing SO at “syn” frequency. (**b**) The response of SO (red) when the external frequency is varied (black). The slow increase of “ext” is the instrumental response.

Investigating the effects of external vibration is important for understanding the origin of SO and its response. A single-frequency, mechanical vibration was applied to the solution (Fig. 3a). When the external frequency was varied in the neighborhood of the synchronized frequency (Fig. 3b, black line), SO was not affected at all (Fig. 3b, red line). Also, SO did not resonate when the external frequency matched, exhibiting its immune nature to external stimuli. We repeated the experiment over frequencies ranging from 3 to 50 Hz and obtained the same result. We also confirmed that applying the external vibration to a stable dispersion (not oscillating) did not induce SO. Thus, SO is not caused by external vibration.

### Power-law noise

**Figure 4:**
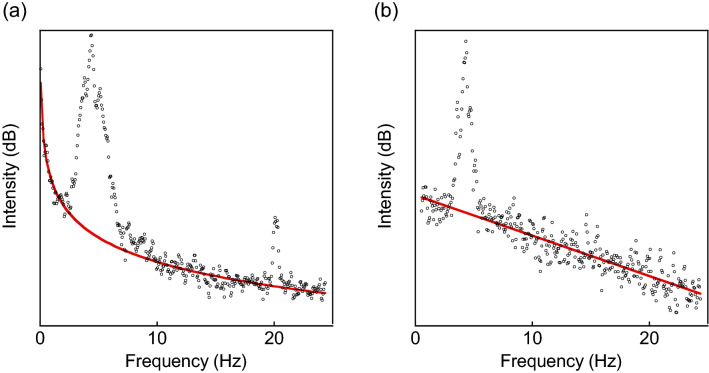
Powe-law noise. (**a**) A power spectrum of SO shown in Fig. 1b. The red curve indicates 1/f^20^ noise. (**b**) A power spectrum of a dispersion that does not show SO. All non-SO dispersions have a linear baseline.

Although SO was observed quite reproducibly, it was absent in some samples despite our consistent experimental procedure. We have noted that, when the sample does not synchronize, the baseline of a power spectrum becomes linear (Fig. 4b), whereas when it synchronizes, the baseline always exhibits $\frac{1}{{f}^{n}}$ dependence (Fig. 4a. In this case, n = 20). The power-law spectrum is known to be caused by complex heterogeneous networks in solution^19^. We conjecture that SO and the power-law noise share the same origin that is related to the thermal motion of heterogeneous CNT networks.

## Discussion

The CNT concentration in this study is 0.15 wt%, which is likely to be below the percolation threshold. Due to its small mass and high elasticity, a single CNT has vibrational resonance frequencies on the order of 100 MHz in a vacuum^20,21^ and 0.5 MHz in water^22^. It is clear that the observed oscillation at 20 Hz is not a synchronization of individual CNTs. On the other hand, we have observed that dense CNT aggregates behave as solid particles, executing Brownian motions when they are a few μm in size and becoming stationary as they grow larger. Thus, an oscillating unit should be somewhere in between these extremes and consist of a sparse CNT network cluster. The linkage is provided by the VDW interaction and the dye-bridging effect at the surfactant-free area.

The synchronized peak has a very narrow width, indicating a small drag by the solvent viscosity. Other than the nanometer-scale diameter of each CNT, the network cluster should be sparse enough to achieve a small drag.

The power-law noise suggests that there are many CNT network clusters of various sizes and possibly different structures, each executing slow dynamics. Then, the observed oscillation is a result of the synchronization of these clusters. We observed SO everywhere over the entire viewing area of 1 cm^2^. This means that these network clusters are interacting over a macroscopic scale.

**Figure 5:**
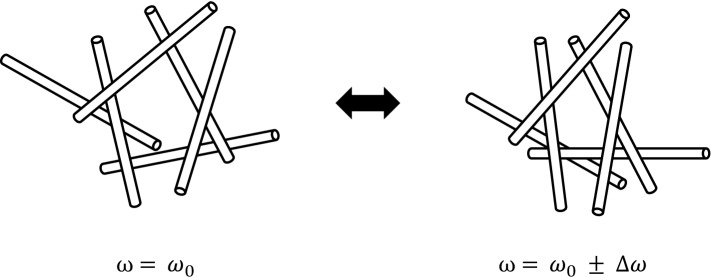
CNT cluster with variable linkages. A slight shift of the position and angle of each linkage modifies a network structure of the sparse CNT cluster, resulting in a change in its oscillating frequency.

How the sparsely linked, CNT network clusters attain synchronization is still not clear. The Kuramoto model is purely mathematical, and there is no reason to assume that the trigonometric interaction exists between the CNT clusters in reality. Synchronization is often accompanied by feedback mechanisms. It should be noted that the linkage provided by the desorption/adsorption of surfactant/dye molecules is a characteristic feature of the present network. Such a thermodynamically driven, dynamical linkage implies that the structure of two crossing CNTs can change: i.e., the linkage position can slide along the CNT axis and the crossing angle can be altered. Since the thermal dynamics of a single CNT in solution is known to depend on its length^23^, the characteristic oscillating frequency of the cluster may be highly correlated with its structure (Fig. 5). Thus, these freedoms allow the structure of CNT clusters to adjust itself to the dynamics of surrounding clusters. The inter-cluster interaction may be transmitted by other CNTs not belonging to any clusters or the dye aggregates bridging them. A network connected by variable linkages may play an important role in the feedback.

## Methods

### Sample preparation

A controlled concentration of aqueous Triton X-100 (Molecular Biology Grade, Calbiochem) solution was used as a solvent. For external vibration studies, it was 0.525 wt% Triton X-100. A precursor 0.30 wt% CNT dispersion was made by sonicating (250W bath-sonicator) CNT (NanoLab, PD15L520) in the solvent for 7 min. Immediately before the measurement, the CNT precursor dispersion was mixed with an equal volume of 500 μmole/L rhodamine 6G (Fujifilm) solution by gentle shaking so that the final concentrations became 0.15 wt% CNT and 250 μmole/L rhodamine 6G.

### Power spectrum

The CNT-dye solution was observed by an epi-fluorescence microscope (Eclipse, Nikon), with an Hg lamp, × 60 objective (NA = 1.49) mounted on a vibration isolating table. The excitation wavelengths were set to 510 < ${\lambda }_{ex}$ < 560 nm and the emission wavelengths 590 nm < ${\lambda }_{em}$^12^. The real image was formed directly on the 2D position-sensitive detector (S5991-01, Hamamatsu Photonics). The analog output was directed to the spectrum analyzer (SR760, Stanford Research Systems) to perform FFT in real time.

### External vibration

A frequency-variable motor (BXM230-A2, Orientalmotor) was mounted on the microscope stage directly next to the solution cell and driven with a free load. Using a simple dye solution, we have confirmed that the motor induces vibrations only at the harmonics of the applied frequency.

## Supplementary Information

Supplementary Video 1.

## Data Availability

The data that support the findings of this study are available within the paper and Supplementary information. Additional relevant data are available from the corresponding author upon reasonable request.
